# Concentrations of Essential Trace and Toxic Elements Associated with Production and Manufacturing Processes in Galician Cheese

**DOI:** 10.3390/molecules27154938

**Published:** 2022-08-03

**Authors:** Emanuel Felipe de Oliveira Filho, Marta Miranda, Tania Ferreiro, Carlos Herrero-Latorre, Pierre Castro Soares, Marta López-Alonso

**Affiliations:** 1 Department of Veterinary Medicine/UFRPE, Rua Dom Manoel de Medeiros, Dois Irmãos, Recife 52171-900, Brazil; felipe130188@gmail.com (E.F.d.O.F.); pcastro.pe@gmail.com (P.C.S.); 2 Department of Animal Pathology, Faculty of Veterinary, University of Santiago de Compostela, Campus Terra, 27002 Lugo, Spain; marta.lopez.alonso@usc.es; 3 Department of Anatomy, Animal Production and Clinical Veterinary Sciences, Faculty of Veterinary, University of Santiago de Compostela, Campus Terra, 27002 Lugo, Spain; 4 Technological Platform: Aula de Productos Lácteos y Tecnologías Alimentarias, University of Santiago de Compostela, Campus Terra, 27002 Lugo, Spain; tania.ferreiro@usc.es; 5 Research Institute on Chemical and Biological Analysis, Analytical Chemistry, Nutrition and Bromatology Department, Faculty of Sciences, University of Santiago de Compostela, Campus Terra, 27002 Lugo, Spain; carlos.herrero@usc.es

**Keywords:** cheese, essential trace and toxic elements, chemometric analysis, ICP-MS, Galicia

## Abstract

The objective of this study was to determine the trace element composition and the toxic metal residues in Galician cow’s milk cheese produced in different systems (artisan, industrial, and organic). Fourteen elements (As, Cd, Co, Cr, Cu, Fe, Hg, I, Mn, Mo, Ni, Pb, Se, and Zn) were determined in 58 representative samples of Galician cheeses by inductively coupled plasma mass spectrometry. The toxic elements were present at low concentrations, similar to those reported for other unpolluted geographical areas. The essential elements were also within the normal range in cheeses. There were no statistically significant differences between smoked and unsmoked cheeses for any of the elements. Chemometric analyses (principal component analysis and cluster analysis) revealed that the industrial cheeses produced in Galicia using the milk from intensive dairy farms were different, in terms of elemental content, from artisan and organic cheeses, in which the elemental contents were similar.

## 1. Introduction

Cheese has long formed part of the human diet. In ancient times, cheese was primarily a concentrated form of milk with the advantages of high protein and fat contents that made it a nutritious and energetic food. It also has the advantage of a longer storage duration than fresh milk. Today, the main reasons for cheese consumption are mainly associated with its manifold uses in the kitchen. In fact, technological advances have led to a multitude of different types of cheese being available on the market that vary in flavour, raw material, texture, and other organoleptic properties. Moreover, cheese is a rich source of essential nutrients such as proteins, amino acids, bioactive peptides, fat, and fatty acids. In fact, the conjugated linoleic acid and sphingolipids present in cheese can exert anti-carcinogenic activity [1]. In addition, cheese is a good source of vitamins, minerals, and elements such as calcium. So, cheese consumption helps maintain strong bones and teeth, but also has positive effects on blood pressure and can help with weight loss when included in a low-energy diet.

The nutritional and organoleptic properties of cheese are highly dependent on the raw material used (mainly the milk) but also on the manufacturing process. The fatty acid profile of cheese is known to be closely related to dairy feed (with grazing of cows having a strong influence [2]), and several sensorial properties are also closely linked to the manufacturing process (raw vs. pasteurized milk [3]). However, information regarding the influence of the trace element concentrations and toxic metal residues on the type of milk and the manufacturing processes of cheese is scarce. Recent studies have indicated important differences in the mineral profile of the raw milk depending on the dairy management process. For example, it has been reported that organic milk contains less iodine than conventional milk [4,5,6] due to the lack of routine mineral supplementation in feed and the less frequent practice of dipping teats with iodine-based disinfectants in organic farming systems [7]. Differences in industrial and artisan manufacturing processes may also influence the concentrations of elements in the cheeses. The concentrations of some elements can be increased by the release of metals from containers and tools, with which milk and intermediate products come into contact [8,9]. For example, the direct and prolonged contact between acidic food and stainless-steel equipment during manufacturing processes can lead to significant amounts of nickel (Ni) and chromium (Cr) leaching into the product [10].

Galicia (NW Spain) has a long tradition of cheese-making and is the leading manufacturer of cow’s-milk cheeses in Spain, with approximately 40% (more than 250 million L per month) of the total Spanish cow’s milk production being generated in the region [11]. Galician cheeses represent one quarter (more than 5.4 million kg) of the total annual production of cow’s-milk cheeses in Spain. Although there are several recognized Protected Denominations of Origin (PDOs) for cheeses, all Galician cheeses are soft or semi-soft cheeses mainly produced from rennet curd with a short ripening time (7–60 days), giving them quite similar organoleptic characteristics. In the past, Galician cheeses were generally elaborated in artisanal processes using raw milk from pastured cattle [12], but nowadays most are produced by an industrial manufacturing process using milk from intensive production systems. However, there is still a market for handmade artisan cheeses (produced directly on farms at a familiar scale and highly appreciated by consumers), which are sold at local markets and in delicatessen stores. The production of organic artisan cheese in Galicia has grown continuously in the past few years, maintaining traditional practices but incorporating organic regulations (prohibiting or minimizing the use of chemicals such as fertilizers or mineral supplements) [13]. Artisan and organic cheeses are produced using raw milk from pasture-fed cows, which gives the cheeses their particular organoleptic characteristics as well as a heart-healthy fat profile. Although not previously studied, artisan cheeses made from milk from pasture-fed cows may have characteristic mineral profiles different from those of cheese made under industrialized manufacturing processes with milk from more technologically-based farms where cows are fed intensive diets with mineral supplements. 

The objective of this study was to evaluate the trace element composition and the toxic metal residues in Galician cow’s milk cheese in relation to the production system/manufacturing process (artisan, industrial, and organic) used.

## 2. Material and Methods

### 2.1. Sample Collection

Fifty-eight representative samples of Galician cheeses were analysed in this study. The European Union applies quality schemes with geographical indications and including traditional specialties (such as PDO) to promote and protect the quality of certain agricultural products and foodstuffs. In the case at hand, all samples were whole cheeses belonging to one of the following PDOs: Arzúa-Ulloa, Tetilla, and San Simon da Costa. From January–February 2019, representative samples of industrial (*n* = 30) and organic (*n* = 10) Galician cow’s-milk cheeses were obtained from local supermarkets and delicatessen stores, while artisan (*n* = 18) samples were purchased directly from producers. Most of the samples were made from pressed white curd, except 10 samples of the industrial San Simon da Costa PDO cheese, which undergoes a smoking process after elaboration [14]. The samples, packed in closed polyethylene bags, were immediately refrigerated, transported to the laboratory, and stored at −20 °C until analysis.

### 2.2. Sample Preparation

For determination of essential elements and toxic metals, 1 g of a homogenate of various subsamples of the cheese were acid digested in 5 mL of 69% concentrated nitric acid (TMA, Hiperpure, PanReac, Barcelona, Spain) and 2 mL of 33% *w*/*v* hydrogen peroxide (PanReac, Barcelona, Spain) in a microwave-assisted digestion system (Ethos Plus, Milestone, Sorisole, Italy). Digested samples were transferred to polypropylene sample tubes and diluted with Milli-Q ultrapure water to yield a final volume of 15 mL. Additional processing was required for determination of iodine (I) by treating the samples after the high temperature alkaline extraction procedure [15] with a mixture of tetramethylammonium hydroxide 25% (*w*/*v*) in water.

### 2.3. Sample Analysis

The concentrations of the essential elements cobalt (Co), Cr, copper (Cu), I, iron (Fe), manganese (Mn), molybdenum (Mo), Ni, selenium (Se), and zinc (Zn) as well as the toxic metals arsenic (As), cadmium (Cd), lead (Pb), and mercury (Hg) were determined by inductively coupled plasma mass spectrometry (ICP-MS, VG Elemental PlasmaQuad SOption equipped with a micromist low-flow nebulizer, Agilent Technologies, Tokyo, Japan), following previously established operational conditions [4,6]. All of the samples were analysed in triplicate, and the concentrations of essential elements and toxic metals in the samples are expressed as µg/kg wet weight (*w*/*w*).

### 2.4. Analytical Quality Programme

An analytical quality control programme was applied throughout the study by including blank samples and certified reference material (CRM) with the samples during analysis (Table 1). The blank values were subtracted from the sample readings, and the limits of detection of the method were calculated as 3 times the standard deviation of the reagent blanks (9 samples). The limits of quantification, expressed as a concentration in the cheese, were calculated based on the sample weight and the dilution. In all samples, mineral concentrations were above the limits of quantification except for Hg, residues of which were negligible in all samples. Analytical recovery, determined by analysis of CRM NIST SRM−1549 (Non-Fat Milk Powder) together with the samples, showed an acceptable level of agreement between the measured and certified values (Table 1).

### 2.5. Statistical and Chemometric Analysis

An *X*_58×13_ matrix was constructed for analysis of the data, with the rows corresponding to the 58 cheese samples and the columns to the contents of the 13 essential elements and toxic metals determined by ICP-MS. Data normality was checked using the Kolmogorov–Smirnov test. The data were not normally distributed and were therefore log-transformed before analysis. The potential influence of the smoking procedure on the trace and toxic element concentrations in the industrial San Simon da Costa cheese against the other industrial samples from other origins was evaluated by the Student’s t-test. Differences in the concentrations of essential elements and toxic metals in artisan, industrial, and organic cheese were evaluated by one-way Anova and post-hoc Tukey tests. All of the statistical analyses were performed using IBM SPSS for Windows v.27 (IBM Corporation, Armonk, NY, USA).

Chemometric analysis of the data was carried out to test the potential influence of the cheese manufacturing process (artisan, industrial, and organic) on the essential and toxic elemental contents. Two unsupervised display chemometric techniques, Principal Component Analysis (PCA) and Hierarchical Cluster Analysis (HCA), were used for this purpose. PCA was applied first to visualize the 13-dimensional data matrices in a reduced dimension, thereby preserving the maximum data variance [16]. HCA (usually applied in combination with PCA) was then performed to establish clusters of similar samples (or variables) based on the Euclidean distance between them as a similarity measure. The final result produced by HCA is a graphical tree diagram called a dendrogram, which is a two-dimensional plot of the sample similarities in the 13-dimensional space of the variables. For both PCA and HCA, original variables were autoscaled prior to multivariate analysis to prevent any influence of the different sizes of mineral variables in the chemometric study. The autoscaling procedure (each value was substituted by a new value obtained by subtracting the mean of the variable and dividing it by the standard deviation) produced new variables of the same size, with zero mean and unit variance [16]. All chemometric analyses were carried out using the software package Statgraphics Centurion XVI v.16.1.15 (Statistical Graphics Corporation, Rockville, MD, USA).

## 3. Results and Discussion

### 3.1. Trace Elements and Toxic Metals Content in Cheese Samples

As described in the Section 2.1, in some parts of Galicia, the cheese undergoes a smoking process. Therefore, two different types of industrial Galician cheeses are available: cheeses made from pressed white curd, and cheeses that are smoked after being aged. Therefore, the first step of the data analysis was to evaluate whether the smoking process influences the elemental content of the samples. The concentrations of essential and toxic metals in smoked and unsmoked industrial cheese are compared in Table 2. No statistically significant differences between smoked and unsmoked cheeses were found for any element. Thus, (i) the potential increase in the metal content in the product due to smoking remains in the rind (not generally eaten) and does not migrate to the part of the cheese that is eaten, and (ii) the absence of significant differences in the metal content implies that both types of cheese belonged to the industrial group, and they were subsequently considered a single group. Therefore, for subsequent statistical and chemometric analyses, the three groups established—artisan, industrial, and organic—were considered as described above.

**Figure 1:**
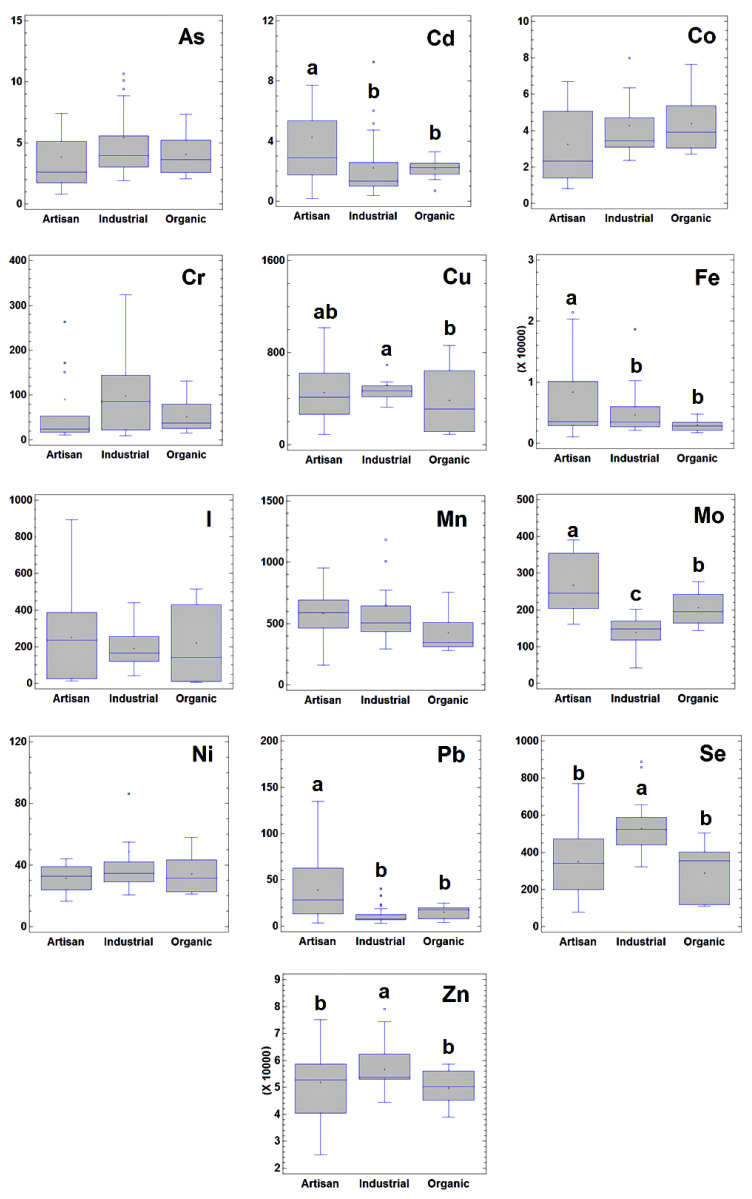
Box and Whisker plot showing concentrations of essential trace and toxic elements in Galician cheese (expressed as µg/kg wet weight) according to the type of manufacturing process (artisan, industrial, or organic). Different letters indicate statistically significant differences between groups (*p* < 0.05).

The results of a univariate study of the concentrations of essential elements and toxic metals in Galician cheese in relation to the manufacturing process are presented as Box and Whisker plots (Figure 1). Overall, toxic metal residues were low and similar to those described elsewhere (Table 3), corresponding to geographical areas with low level of environmental exposure. Essential element concentrations were also within the normal range for cheeses. Moreno-Rojas and coworkers [17] analysed 50 varieties of cheeses made in Spain and reported average levels of toxic metals similar to those determined in the present study. Nevertheless, cheeses made from cow’s milk from polluted regions had higher levels of Pb or As [18,19], whereas in all of these studies the Cd levels were generally low and similar to those in unpolluted areas [19]. 

Statistically significant differences between the cheeses were found regarding most of the main toxic and essential elements, depending on the type of manufacturing process (Figure 1). Overall, two main patterns were observed. On the one hand, the industrial cheese contained significantly higher levels (*p* < 0.05) of the essential trace elements that are routinely supplemented in intensive dairy farming: Cu, Se, and Zn [20,21]. Although there were no statistically significant differences in the Cr concentrations between the industrial cheeses and the artisan and organic cheeses, a tendency for a slightly high Cr concentration was detected in the industrial products. This finding can be explained by the contact between the milk/raw cheese dough and stainless-steel equipment (with a Cr content up to 20%) during the industrial manufacturing process [10]. On the other hand, the artisan cheeses contained significantly higher concentrations of toxic metals (e.g., Cd and Pb), compared to the industrial and organic cheeses. In addition, the concentrations of Fe and Mo were also significantly higher in the artisan cheeses than in the other cheeses. This finding may be related to the ingestion of soil by cows during grazing. Soil ingestion constitutes an important route of exposure in cattle to contaminants that are not geochemically or biologically mobile [22].

**Table 3:** Concentrations of essential trace and toxic elements (expressed in µg/kg wet weight) in cheese reported in previous studies.

Country	Type of Cheese	Co	Cu	Cr	Fe	Mn	Mo	Ni	Se	Zn	As	Cd	Hg	Pb	Ref
Italy	White cheese	-	-	331	-	-	-	347	-	-	-	180	-	750	[23]
Turkey	Kaşar cheese	-	700	-	4200	-	-	-	-	37,700	-	1.8	-	86.0	[24]
Turkey	Herby cheese	1300	3100	3300	40,800	-	-	2400	-	-	-	100	-	5200	[25]
Turkey	White cheese	-	-	-	-	-	-	-	160	-	30	120	-	920	[26]
	Kaşar cheese	-	-	-	-	-	-	-	280	-	20	30	-	1100	
	Tulum cheese	-	-	-	-	-	-	-	430	-	70	50	-	610	
	Lor cheese	-	-	-	-	-	-	-	ND	-	70	20	-	450	
Spain *	various cheeses	-	-	-	-	-	-	117.3	-	-	-	4.70	16.10	32.77	[17]
Saudí Arabia	White cheese	-	160	-	7630	500	-	-	-	7190	-	140	-	470	[27]
France *	Comté cheese	-	13,520	-	-	-	-	-	-	48,140	-	1,3	-	47	[28]
Hungary	Trappista cheese ^1^	-	455	314	7258	367	-	929	-	23,890	ND	ND	-	126	[29]
	Trappista cheese ^2^	-	695	528	7979	418	-	903	-	19,246	ND	ND	-	149	
Turkey *	White cheese	40	280	90	-	30	-	120	170	8910	-	40	-	140	[9]
Lebanon	White cheese	27,200	480	1.1	2400	180	50	70	120	21,500	7.0	0.14	1.006	20.7	[30]
Egypt	Fresh cheese	-	3250	-	-	-	-	-	-	-	-	240	32	610	[31]
Egypt	Cheese	-	87	-	3930	-	-	-	-	8590	-	90	-	430	[32]
Spain *	Genestoso cheese	-	2050	-	3960	450	-	-	-	21,240	-	-	-	-	[33]
South Korea	Cheese	-	-	-	-	-	-	-	-	-	-	870	-	5640	[34]
Italy	Asiago cheese	-	1760	-	1480	-	-	-	830	26,840	-	-	-	-	[35]
Iran	Cheese	-	428.0	-	-	-	-	-	1.68	586.0	-	1.25	-	14.5	[36]
México *^,‡^	Oaxaca cheese	-	20	10	-	-	-	30	-	180	170	-	-	50	[18]
	Ranchero cheese	-	20	20	-	-	-	10	-	740	160	-	-	110	
	Curd cheese	-	20	30	-	-	-	2	-	690	70	-	-	20	
Brasil	Coalho cheese	-	5900	11,000	9000	3100	-	-	-	43,000	-	-	-	-	[37]
	Minas padrão cheese	-	6100	1300	10,000	2200	-	-	-	40,000	-	-	-	-	
	Minas frescal cheese	-	6700	1200	8000	2300	-	-	-	39,000	-	-	-	-	
Italy	Cheese	-	1070	50	2230	-	-	-	-	5240	-	2	40	70	[38]
Romania *	Ripened cheese	1120	-	-	-	710	-	-	-	70,640	90	-	-	-	[39]
Greece	Graviera Cheese	80.0	800.0	650.0	-	-	100.0	430.0	110.0	-	25.0	5.2	-	30.0	[40]
Greece	Cheese	-	270.0	27.8	2090	389.0	-	78.9	125.0	16,000	-	0.150	0.34	3.171	[41]
Slovak Republic *	Oštiepok Cheese	-	10,000	1000	14,100	700	80	-	440	23,200	-	-	-	-	[42]
Slovak Republic	White cottage cheese	30	110	170	1750	680	-	90	-	1800	-	-	-	-	[43]
Romania *	Cheese	-	2300	-	-	-	-	-	-	3200	-	3.3	-	240	[44]
Denmark *	Blue cheese	-	-	-	2680	160	-	-	110	27,300	-	2	-	13	[45]
Georgia ^‡^	Imeruli cheese	13	1.261	35	69,090	896	289	11	1.003	75,860	-	2	-	121	[19]
	Sulguni cheese	30	2.463	790	101,100	2.348	401	26	3060	124,800	-	7	-	258	

* Dry matter; 1—non-polluted green area; 2—Highway area; ND—non detected; ^‡^—polluted area.

In fact, in organic and non-intensive grazing cattle, soil ingestion during grazing strongly influences the trace element status of the milk [6]. The different patterns in the organic and artisan cheese, despite the similar livestock production systems (based on pastures), may be related to a lower exposure to toxic metals in this production system. The use of chemicals has been shown to represent the main source of exposure to toxic metals in animals, and in organic farming the use of chemicals is strictly limited [13]. In conventional agricultural systems, traces of Cd in phosphate fertilizers are known to represent one of the main sources of Cd exposure [46].

### 3.2. Chemometric Analysis

**Figure 2:**
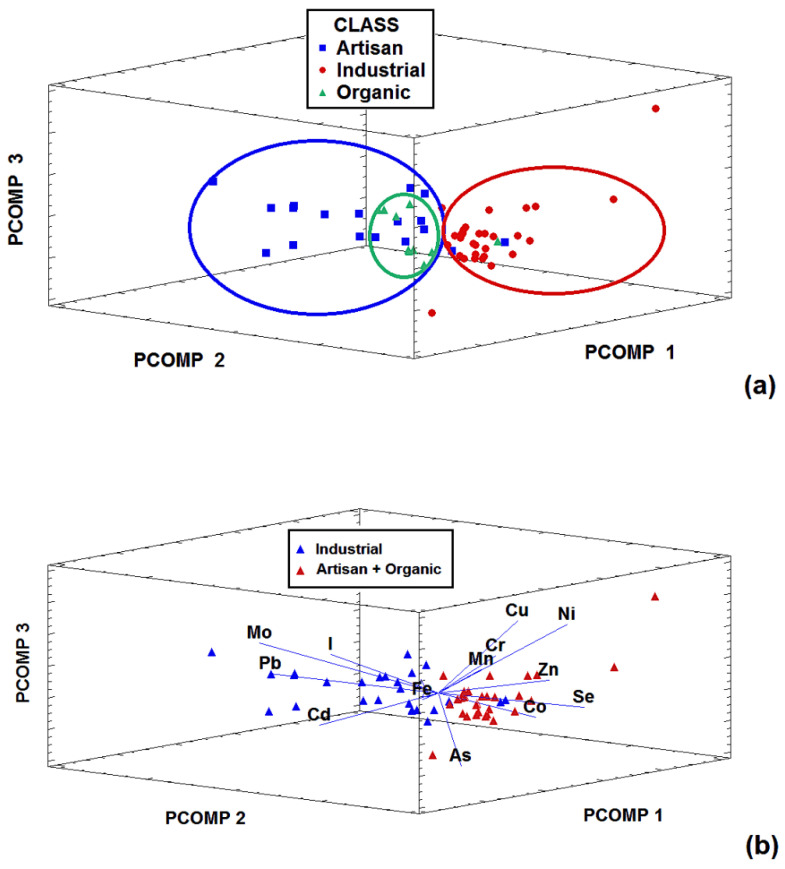
Principal component analysis (PCA) score plot for (**a**) the cheese samples and (**b**) cheese samples plus variables according to their type on the space defined for the first three principal components representing 50.5% of the total variance.

Multivariate chemometric techniques were used in an attempt to identify situations in which more than a single factor is involved. The main advantage of using these techniques is that a more realistic picture of the chemical problem is obtained as opposed to when each variable is studied separately [47]. In addition, the multivariate approach provides useful information about the relationships between samples, between variables, and between variables and samples. Thus, in the present work, two previously described display multivariate chemometric techniques, PCA and HCA, were used to explore the latent sample-variable structures and relations in the data set. A principal component analysis was conducted on the *X*_58×13_ matrix after autoscaling. The visualization of the score-plot of the samples (see Figure 2a) in the space defined by the first three principal components (accounting for 50.5% of total data variance) yielded interesting results regarding the different groups of cheese. As can be seen in the figure, industrial samples constituted a homogeneous group, separate from the artisan samples, which also included organic samples as a subgroup. This finding can be explained by considering that industrial cheeses are generally elaborated following technological procedures that tend to homogenize the characteristics of the product. By contrast, in the group of artisan cheeses, the production system used does not apply standardized procedures and in each case is carried out following the traditional processing system in the area, which explains the higher heterogeneity in this group. The inclusion of the organic group in the artisan cluster was also not surprising. In Galicia, artisan cheeses are generally produced on small, family-managed farms using milk produced in a pasture-based livestock system. Thus, artisan production is very similar to organic production because the use of mineral-supplemented feed and other chemicals is very limited on these small farms (for economic reasons) and the production procedures are comparable to those used by organic-certified producers. From an examination of the samples in the PCA-biplot, in which the samples and variables are represented together in the principal components space (Figure 2b), it can be concluded that Cd, Pb, and Mo are clearly associated with the artisan group, while the remaining elements are more closely associated with industrial cheeses, except for I, which interestingly appears in the same area of the PC-space where the organic cheeses are located. This is a surprising finding because organic milk is generally low in I [4,5]; therefore, it might be expected that the organic cheeses produced from this milk would also be low in I. However, when the I levels were examined in greater detail (See Figure 1), it was found that (i) the mean I contents were similar, increasing from the organic to the industrial and the artisan groups, and (ii) the variability in the I content was high for artisan and organic samples, but low for industrially produced cheeses. The explanation for this finding is also related to the production process: both artisan and organic producers add iodized salt to the cheeses. Iodized salt is widely available and commonly consumed in Galicia as part of an iodine prophylaxis policy aimed at preventing iodine deficiency disorders, including goiters, previously endemic in the region. On the other hand, industrial cheeses are produced under established manufacturing protocols using common, limited amounts of salt. Comparable levels of I, ranging from 132 to 468 µg/kg, have been reported for organic cheese produced in Norway [48].

**Figure 3:**
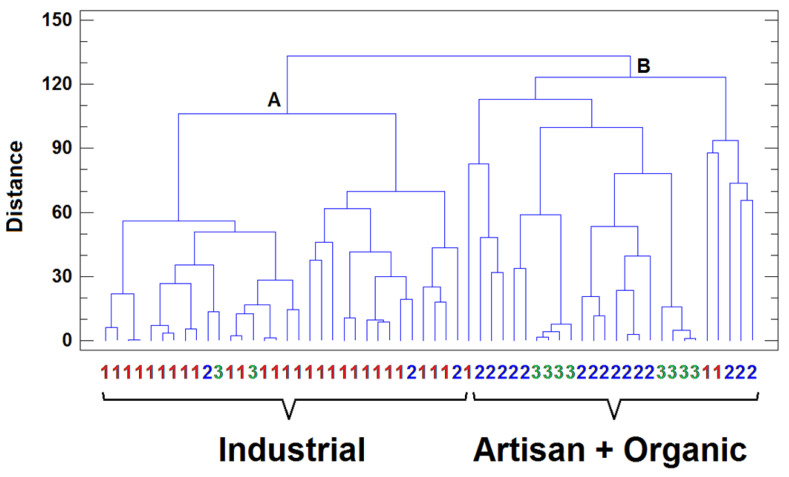
Hierarchical cluster analysis (HCA) dendrogram of milk samples: 1, industrial; 2, artisan; 3, organic.

**Figure 4:**
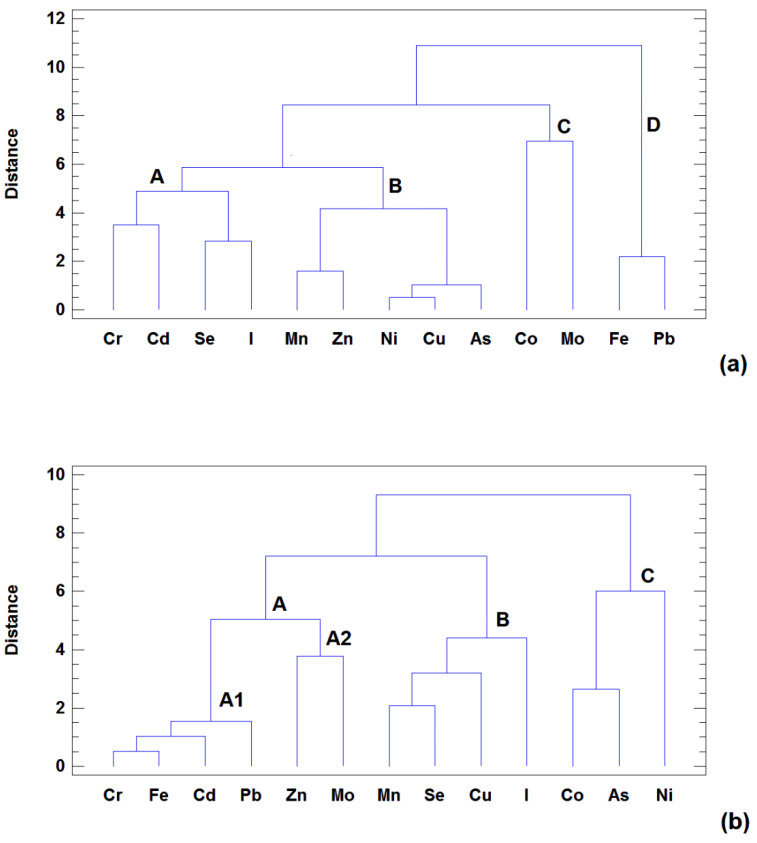
Hierarchical cluster analysis (HCA) dendrograms of the variables based on Euclidean squared distance and Ward method: (**a**) industrial samples and (**b**) artisan-organic samples.

The second step in the chemometric study was to apply HCA to the X-matrix after autoscaling. In this case, the similarity/distance between the samples (or variables) was calculated using the Euclidean distance, while the Ward method was used to establish the clusters. The result obtained when the samples are clustered under the mentioned conditions is shown in Figure 3. Two main clusters of samples were revealed. From the left, the first cluster (A) comprises most of the industrial samples, while the second cluster (B) includes artisan and organic samples. These results are consistent with those obtained by PCA and confirm the group arrangement of cheese samples based on the metal contents. To determine whether the relationships between the metals are similar for the industrial and for the artisan plus organic samples, HCA was applied separately to both groups for the variables (using the same distance measurement and agglomerative procedure previously described). The dendrograms obtained are shown in Figure 4a,b. According to the clusters obtained, very different patterns of relationships between variables for both groups were observed. In the industrial samples (Figure 4a), made with milk from intensive farm systems, three main clusters were identified. The first cluster (A), including elements such as I, Se, Cd, and Cr, is related to the hygiene-management practices in the intensive milk production system and to the industrial cheese elaboration process.

The first subcluster in this variable-group comprises Se and I. Selenium treatments are commonly used in intensive dairy systems to increase the fertility of the cows, as well as to minimize placental retention and the incidence of mastitis and metritis [49]. On the other hand, iodine-based disinfectants such as non-rinsing detergents and antiseptics are commonly applied to contact surfaces to eliminate pathogenic microorganisms that can cause diseases leading to economic losses in the livestock and food industries. Washing and dipping the teats before milking affects the iodine content of milk and therefore the cheese [50]. The presence of Cd and Cr in the other subcluster is associated with the use of stainless-steel equipment in the industrial fabrication of cheese [51]. Cluster B included Cu, Zn, and Mn, all of which are associated with feeding as they are all routinely added to the concentrate feed in the dairy industry [20,21]. The elements in clusters C (Co and Mo) and D (Fe and Pb), which are well separated from clusters A and B, indicate that these groups are associated with the soil [21]. Thus, there are three factors involved in the metal contents of the industrial cheeses: the management of intensive milk production and the industrial elaboration of cheese (cluster A), feeding practices (cluster B), and soil influence (clusters C/D). 

The artisan-organic group of cheese (Figure 4b) showed a completely different pattern of metal contents. In this case, Cr and Cd appeared linked in subcluster A1 with a high level of similarity to Fe and Pb, which originate from the soil. Thus, for the artisan-organic group, the levels of Cr and Cd were not associated with the industrial elaboration of the cheese, but with cows ingesting soil during grazing. In these production systems, in which mineral supplements are not added to the diets (organic farms) and minerally supplemented concentrate feed is only occasionally used (pasture-managed farms), the consumption of soil during grazing represents the main source of exposure to trace elements [21], which occur in soil at much higher concentrations (up to four orders of magnitude) than in vegetables [52]. The inclusion of Zn and Mo (subcluster A2) in the same cluster also contributes to this explanation. Cluster B includes several elements that are presumably related to feed (I, Se, Cu, and Mn). 

Although artisan-organic cheese is elaborated using milk from pasture-fed cows (which are not routinely administered mineral supplementations), the occasional use of minerally supplemented concentrate as complementary feed during the winter in the small family dairy farms that produce artisan cheese may explain our findings. In this case, Se and I are included within the “feed” group as both elements are included in the mineral premixtures, but unlike in intensive dairy farming, they are not used in hygiene-management practices. Finally, we identified a third cluster, C, including elements typically associated with the soil (Co, As, and Ni). We do not know why these elements appear separately from Fe or Cr, which are clearly associated with the soil and with which they have been found to be closely related in previous organic farming studies conducted by our research group [6,21,52]. It is possible that some difference in the grazing management between the organic and small conventional farms producing artisan cheese may explain this separation. 

## 4. Conclusions

Galician cheeses were found to have a low level of toxic metal residues and normal levels of essential trace elements. The smoking process does not affect the elemental content of the cheeses. The chemometric analysis revealed the following: (i) the industrial cheeses produced in Galicia using milk from intensive dairy farms are different, in terms of their elemental contents, from artisanal and organic cheeses. (ii) The artisanal and organic cheeses are similar in terms of their element profiles; therefore, both groups were superimposed in the multidimensional space of the variables. Thus, the artisanal cheeses are similar to organically produced cheese, with respect to their elemental content. Finally, (iii) the relationships between different elements in the industrially produced cheeses can be explained by the influence of the type of milk production, the technological processes of the cheese production, the livestock diet, and the soil. By contrast, in the case of artisan and organic cheese, relationships between variables were limited to the influence of the soil and to a lesser extent the diet.

## Figures and Tables

**Table 1:** Results of the analytical quality control programme applied during determination of the essential trace and toxic elements in cheese by ICP-MS in the present study.

Element	Detection Limit (mg/L)	NIST-1549		
		Certified Level (Mean ± SD; mg/kg)	Level Determined (Mean ± SD; mg/kg)	% Recovery
As	0.020 × 10^−3^	(0.0019) *	0.0019 ± 0.0004	102.1
Cd	0.007 × 10^−3^	0.0005 ± 0.0002	0.0005 ± 0.0001	100.9
Co	0.004 × 10^−3^	(0.0041)	0.0041 ± 0.0009	98.4
Cr	0.009 × 10^−3^	0.0026 ± 0.0007	0.0025 ± 0.0002	94.6
Cu	0.006 × 10^−3^	0.700 ± 0.100	0.661 ± 0.028	94.4
Fe	0.132 × 10^−3^	1.78 ± 0.10	1.93 ± 0.46	110.9
I	0.251 × 10^−3^	3.38 ± 0.02	3.56 ± 0.32	105.0
Mn	0.023 × 10^−3^	0.26 ± 0.06	0.24 ± 0.03	91.4
Mo	0.006 × 10^−3^	(0.34)	0.325 ± 0.011	95.8
Ni	0.018 × 10^−3^	-	-	
Pb	0.002 × 10^−3^	0.019 ± 0.003	0.019 ± 0.002	98.1
Se	0.217 × 10^−3^	0.11 ± 0.01	0.12 ± 0.01	108.1
Zn	0.038 × 10^−3^	46.1 ± 2.2	44.3 ± 2.4	96.2

* in brackets indicative values.

**Table 2:** Concentrations of essential trace and toxic elements (expressed as mean ± standard error in µg/kg wet weight) in smoked (San Simon da Costa) and unsmoked (Arzúa-Ulloa, Tetilla) industrial cheeses.

Element	Smoked	Unsmoked	*p*-Value
As	4.61 ± 0.74	4.16 ± 0.42	0.568
Cd	2.49 ± 0.79	2.07 ± 0.38	0.590
Co	3.30 ± 0.21	4.75 ± 0.53	0.073
Cr	89.4 ± 31.0	101.4 ± 15.7	0.701
Cu	426 ± 19	561 ± 84	0.272
Fe	4196 ± 933	4178 ± 376	0.983
I	156 ± 18	209 ± 23	0.149
Mn	510 ± 26	719 ± 161	0.374
Mo	135 ± 14	140 ± 10	0.764
Ni	29.7 ± 1.9	58.1 ± 11.0	0.079
Pb	10.0 ± 1.7	13.3 ± 2.4	0.369
Se	558 ± 48	514 ± 26	0.383
Zn	56,611 ± 2504	56,737 ± 1740	0.967

## Data Availability

Data are available in the manuscript.

## Sample Availability

Samples of the compounds are not available from the authors.
